# Data-driven, client-centric applied behavior analysis treatment-dose optimization improves functional outcomes

**DOI:** 10.1007/s12519-022-00643-0

**Published:** 2022-11-17

**Authors:** Andrey Ostrovsky, Melissa Willa, Ted Cho, Maxwell Strandberg, Sage Howard, Colin Davitian

**Affiliations:** 1 Social Innovation Ventures, Lewes, DE USA; 2 Kyo, San Francisco, CA USA; 3 UCSF Benioff Children’s Hospital, San Francisco, CA USA; 4 SKF Group, Göteborg, Sweden

**Keywords:** Autism spectrum disorder (ASD), Autistic disorder, Autistic rehabilitation, Autistic therapy, Telemedicine

## Abstract

**Background:**

With increasing numbers of individuals diagnosed with autism spectrum disorder (ASD) and with affirmation of applied behavior analysis (ABA) as an evidence-based standard of care for ASD, there has been a proliferation of agencies offering ABA services over the last several decades. Disagreement exists among ABA providers and health plans that reimburse those providers on the optimal number of hours of ABA services that should be reimbursed. This study aims to understand whether children who receive more hours of ABA therapy achieve better outcomes and to evaluate the impact of the COVID-19-induced shift to telehealth clinical supervision on outcomes.

**Methods:**

A retrospective cohort analysis was performed using data from the Vineland 3 Comprehensive Interview Form to assess function throughout ABA treatment. Paired sample *t* tests, independent sample *t* tests, Cohen’s D, and Pearson correlations were used to determine relationships between Vineland scores and input variables including hours of service and modality of supervision (in-person vs. telehealth).

**Results:**

While statistically and clinically significant improvements in function were observed, children appear to have improved outcomes independent of the number of hours of service received. There were also no significant associations between modality of supervision and Vineland standard scores.

**Conclusions:**

These findings challenge prior research that demonstrated a linear dose–response relationship. By tailoring treatment dosage to the individual client’s needs, providers may be able to better maximize functional progress of the client, to preserve family time, and to utilize health plan dollars more efficiently.

**Supplementary Information:**

The online version contains supplementary material available at 10.1007/s12519-022-00643-0.

## Introduction

The prevalence of autism spectrum disorders (ASD) has increased significantly during the last several decades. Although estimates vary, as of 2016 the ASD prevalence was 1 in 54, with a relative increase of 170% from 2000 when it was 1 in 150 [1–3]. Other reports estimate that the total population of autistic children served by the public education system in the United States has risen to over 828,000 children aged 8–21 years [4].

ASD is a treatable, neurodevelopmental condition that requires a comprehensive and intensive intervention individualized to the child and family’s needs to address social communication, language, play skills, and maladaptive behavior. A growing evidence base suggests that care based on the principles of applied behavior analysis (ABA) is associated with socially significant skill gains for children with ASD [5, 6]. ABA is an intensive, behavioral intervention that seeks to target the defining characteristics of ASD, such as delays in social communication and restricted, repetitive interests, behaviors, and activities [7]. ABA uses behavior modification to reinforce behaviors that an autistic person and their family wish to improve and to decrease behaviors that serve as potential barriers to desired quality of life [6]. Although ABA has demonstrated improvement in outcomes in several reasonably designed studies, efficacious adoption, implementation, and maintenance of interventions for autism are variable in community settings [8].

Despite variability in efficacy in community implementation of ABA, due to increasing numbers of individuals diagnosed with ASD, and affirmation of ABA as an evidence-based standard of care for ASD, there has been a proliferation of agencies offering ABA therapy services over the last several decades. As of 2021, the US autism care market was estimated to be valued at $3.78 billion and was forecasted to have 4.5% average annual growth [9].

The growth in number of ABA providers and the variability in quality of ABA outcomes in the community setting are perpetuating historical efforts by private health insurance plans to limit coverage of ASD care [10]. Disagreement among ABA providers and health plans exists on the optimal number of hours of ABA services that should be reimbursed. Some guidelines recommend that children with ASD have access to at least 100 hours per month of ABA services [5]. ABA providers generally cite these guidelines to advocate health plans to maximize the number of hours to ensure adequate functional progress for the client and to ensure financial sustainability of their organization. While health plans also want to ensure adequate functional progress in their members, they are responsible for sustainable financial stewardship of premium dollars, which requires plans to minimize reimbursement of clinically unnecessary hours and to curb fraud, waste, and abuse.

A National Academies of Medicine report found that treatment intensity was associated with improved outcomes, but the dose–response relationship was unclear, especially at higher doses of treatment and later in the treatment course [5, 11]. More recent studies have begun to add clarity to the treatment-dose response by demonstrating a positive, linear relationship between treatment intensity and outcomes whereby an increase in care hours per week predicted a higher number of mastered learning objectives [12, 13] in children with ASD receiving community-based ABA intervention [14, 15]. However, treatment intensity and duration only accounted for about 60% of the variance found in treatment response. Furthermore, a growing body of evidence suggests that the overall quantity of therapy may be a poor predictor of outcomes [16–18]. Given the lack of clarity in the dose–response relationship at higher doses and given that at least 40% of variation in treatment response is attributable to factors unrelated to treatment dosage, opportunities exist for practice improvement and innovation that allow for more efficient achievement of clinical outcomes.

Technology could be an enabler of practice innovations that personalize care to individual clients so that each client could receive an adaptable and unique treatment dose, thereby reducing treatment response variance and moving away from a one-size-fits-all, 100 hours per month minimum. Given the fragmented nature of the autism services market, few providers have the resources to invest in outcomes collection and analytics tools. Thus, there have been few opportunities for technology-driven innovation and research about how to better align provider and payer goals using more predictable and measurable treatment-dose response of ABA therapy.

One California-based ABA provider with a home-grown population health management tool has been using a novel, data-driven approach to customize the number of service hours to each client. The primary aim of this retrospective cohort study was to understand how a data-driven, client-centric ABA approach would impact the expected treatment-dose relationship for children with ASD.

With the COVID-19 pandemic and the unprecedented growth in telehealth utilization [19], the variation in treatment response to dose may have changed. ABA care delivered through telehealth has preliminary evidence of positive effects [20], but the research remains limited, and the impact of COVID-19 on telehealth has not been adequately studied in the ASD population. A secondary aim of the present study was to evaluate the impact of the COVID-19-induced shift to telehealth clinical supervision on functional outcomes.

## Methods

### Study design

This study was a retrospective cohort analysis using the electronic medical records from Kyo, a California-based ABA provider. The primary hypothesis was that children who received data-driven, customer-centric ABA services would have improved outcomes independent of the number of hours of service they received. The secondary hypothesis was that the modality of supervisory-level care, whether in-person or telehealth, would have no impact on outcomes.

### Intervention

The intervention included provision of ABA services and use of health information technology (HIT) by Kyo. The Kyo care approach employed a developmental, behavioral therapy model that tailored each client’s care program to maximize progress while preserving family time through careful tuning and client-centric, data-driven care plans. Care plans were developed by a board certified behavior analyst (BCBAs) after carefully considering a patient’s learning profile and zeroing in on a targeted amount of therapy using factors such as the patient’s age, language skills, areas of delay, and potential barriers to care. Kyo care team members were trained and reinforced to find the optimal treatment dose for each client. Each client received a unique treatment dose through some combination of behavior technician (BT), program supervisor (PS), and behaviorist (BEH) time. The BEHs are all BCBAs. All direct therapy was provided by BTs in-person for all clients. Supervision and direction of the BTs was provided by BEHs through a combination of in-person and virtual supervision via telehealth. The BEH supervised and directed the BT by observing the BT interacting with the patient and implementing the treatment plan, where the BEH provided feedback to the BT and adjusted the patient’s treatment plan accordingly. Organizational processes and culture reinforced that individual client service hour goals were to be determined based on iterative Vineland scores and the BEH’s clinical assessment. The Kyo Clinical Policy Manual strongly discourages a “more is better” and age-specific minimum hour requirements approach.

One of the processes that ensured high fidelity adherence to the Kyo Clinical Policy Manual was quality assurance checks and care plan reviews throughout the course of treatment between clinical directors and their BCBAs. The Kyo Clinical Policy Manual included a rigorous policy for documenting progress toward care goals and clinical rationale for modifying the care plan. Another process to ensure data-driven, client-centric care was the annual Long-Standing Client Review process for clients with multi-year tenures with Kyo to ensure that they continued to make progress and were receiving the most appropriate mix of supports.

To support its clinical model, Kyo used a commercial-off-the-shelf (COTS) EHR (CentralReach) to record clinical encounters. Kyo also created its own data repository and analytics dashboard that extracted the COTS EHR data to visually inform population health management and individual client care plan refinement. Kyo’s technology provided the Kyo care team with data to enable rigorous adherence to the client-centric processes outlined in the Kyo Clinical Policy Manual. Kyo’s technology also facilitated tracking of each client’s functional score and care plan progress.

### Measurement

The Vineland 3 Comprehensive Interview Form was administered by a Kyo BCBA to each client’s parent and was used to assess function among clients throughout their care journey with Vineland scores generally obtained every 6–12 months. Inclusion criteria for this study required clients to have at least two Vineland scores obtained. Vineland scores entailed a standard Adaptive Behavior Composite (ABCStd) score, which represented overall age-adjusted functioning. Within the ABC standard score were three age-normalized domain scores, including communications (CommStd), daily living (DailyStd), and socialization (SocialStd).

Services were tracked for three types of care team members including BTs, PSs, and BEHs. BEH-level staff were senior clinicians with a master’s degree in Behavior Analysis and were BCBAs. They designed the care program and made updates to the protocol as needed. BCBAs were encouraged to provide in-person consultations when new BTs began work with clients, but otherwise BCBAs were empowered to do supervision virtually. PSs provided mid-level supervision assisting the BEHs with modeling program changes to the behavior technicians. BTs were entry-level clinicians and worked one-to-one with the clients providing the direct therapy, which made up the bulk of the client’s therapy hours.

### Statistical analysis

The study also tracked payer type, which included commercial insurance as well as Medi-Cal, California’s Medicaid program. The data were obtained from a clinical record extracted from Kyo’s data repository and analytics dashboard. Paired sample t tests, independent sample t tests, Cohen’s D, and Pearson correlations were used to determine relationships between ABCStd scores, domain standard scores, and input variables including hours of service; modality of supervision, including in-person and telehealth; and payer type. A *P*-value of 0.05 was used as the threshold for statistical significance. The ABCStd score, CommStd score, SocialStd score, and DailyStd score improvements of 2.0, 2.0, 2.6, and 2.6 were used as thresholds for clinical significance, respectively [21]. The distinction of clinical significance was made from statistical significance to ensure differences detected in the study would have material relevance in the practice setting rather than just in research settings. All data analysis was performed using IBM SPSS Statistics for Windows, version 20 (IBM Corp., Armonk, N.Y., USA).

This study was sponsored by Kyo. The study did not go through an Institutional Review Board because all of the data collection was retrospective, deidentified, and did not pose any risk to participants. The study design, statistical methods, and endpoints were determined collaboratively by the sponsor, its clinical and scientific advisors, independent clinicians and researchers, and study investigators. Statistical analysis was performed by independent statisticians by Social Innovation Ventures (Lewes, DE).

## Results

In total 178 unique clients with a mean age of six years and nine months [range: 23 years; median: 5 years and 2 months ± standard deviation (SD): 4 years] were assessed using a Vineland 3 at two separate times. Data collection occurred from February 2018 through September 2020, and the mean time between assessments was 272.7 days (SD: 133.8 days). Across this population, the mean in-person monthly service hours delivered were BT: 27.53 hours (SD: 15.16 hours), PS: 0.23 hours (SD: 0.78 hours), and BEH: 6.16 hours (SD: 2.68 hours). Additionally, mean monthly service hours delivered remotely (i.e., by telehealth) were BT: 0.71 hours (SD: 2.84 hours), PS: 0.01 hours (SD: 0.05 hours), and BEH: 1.46 hours (SD: 2.31 hours).

**Fig. 1:**
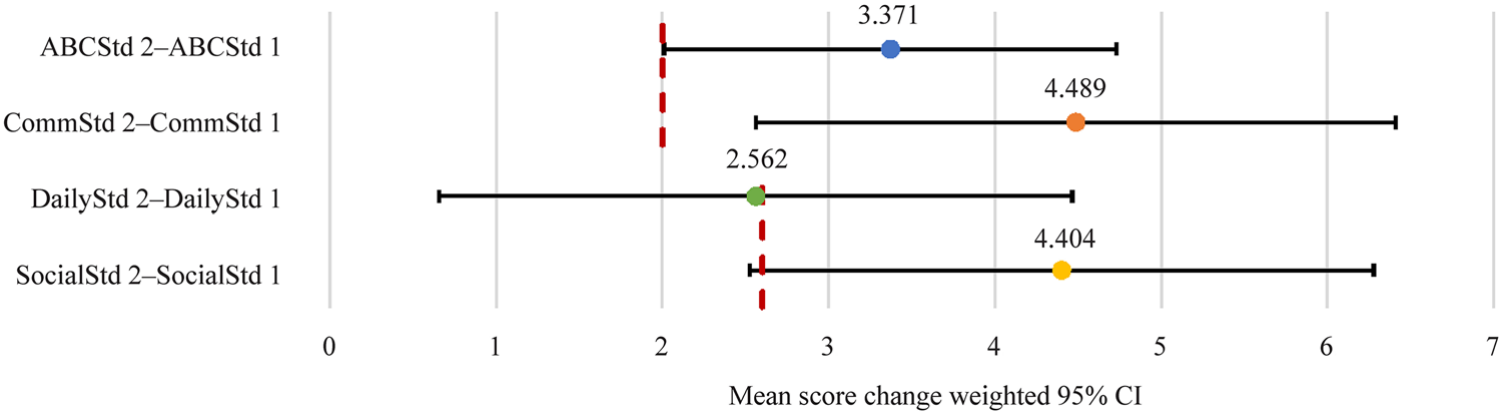
Change in standard scores from time 1 to 2. *ABCStd* standard Adaptive Behavior Composite score, *CommStd* communication domain score, *DailyStd* daily living domain score, *SocialStd* socialization domain score, *CI* confidence interval

All scores, including ABCStd, CommStd, DailyStd, and SocialStd, showed statistically significant improvement from time 1 to time 2 of 3.371 (*P* < 0.001), 4.489 (*P* < 0.001), 2.562 (*P* = 0.009), and 4.404 (*P* < 0.001), respectively (Fig. 1). At the previously stated thresholds for clinical significance (ABCstd = CommStd = 2.0; DailyStd = SocialStd = 2.6), ABCStd and CommStd were shown to have clinically significant improvement, while improvement in SocialStd scores were trending toward demonstrating clinical significance. Given that ASD encompasses a wide spectrum of presentation, these wide confidence intervals are to be expected, making affirmation of clinical significance challenging. With that being said, both the magnitude and direction of the improvement in the SocialStd score are suggestive of clinical significance as well. These findings taken together with a Cohen’s D value of 0.244 are consistent with at least a small effect size (Supplementary table 1).

**Fig. 2:**
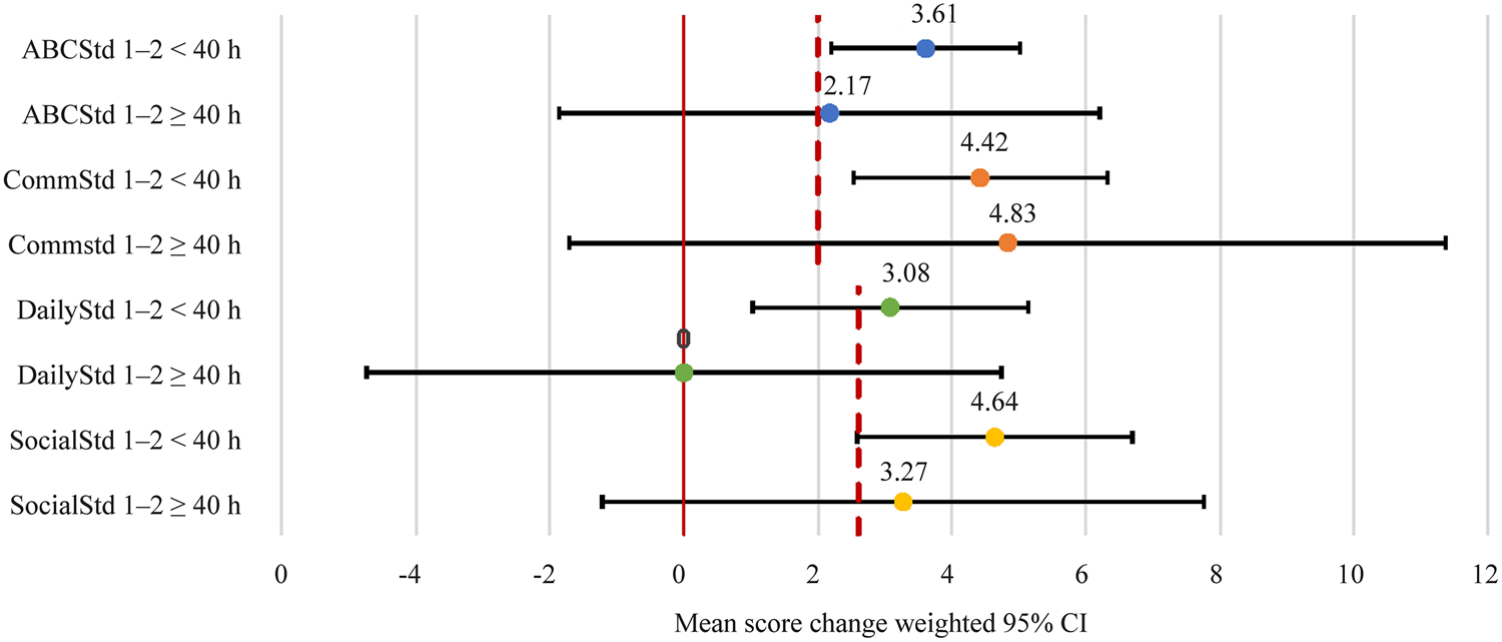
Standard scores relative to hours of service per month. *ABCStd* standard Adaptive Behavior Composite score, *CommStd* communication domain score, *DailyStd* daily living domain score, *SocialStd* socialization domain score, *CI* confidence interval

Children receiving < 40 hours of ABA services per month demonstrated statistically significant improvement (with 95% confidence intervals greater than zero) from time 1 to time 2 in ABCStd as well as in all domain-specific Vineland scores (Fig. 2). Furthermore, improvements in ABCStd, CommStd, and SocialStd in the < 40 hours group were clinically significant as well. However, the improvements in Vineland scores for children with ≥ 40 hours of ABA services per month were not statistically significant, most likely due to the small sample size, which contributed to wide variation rather than being a clinically significant distinction. There were no statistically significant differences in the improvements in ABCStd, CommStd, DailyStd, and SocialStd scores when receiving ≥ 40 hours vs. < 40 hours of ABA services per month with mean differences of −1.448 (*P* = 0.511), 0.414 (*P* = 0.906), −3.081 (*P* = 0.249), and −1.368 (*P* = 0.590), respectively (Supplementary table 2). Of note, the 40-hour threshold was used for analysis because this was the approximate mean of services delivered to participants.

The absence of significant differences in function relative to ≥ 40 hours vs. < 40 hours of ABA services per month is reinforced by the finding that while the children demonstrated significant improvements from time 1 to time 2, there was no significant association between hours of service for BT, PS, and BEH and Vineland standard score improvements (Pearson coefficient magnitudes never being greater than 0.117) (*P* = 0.119) (Supplementary table 3). The lack of significant differences and correlations supports the notion that improvements in Vineland scores were independent of the treatment dose.

**Fig. 3:**
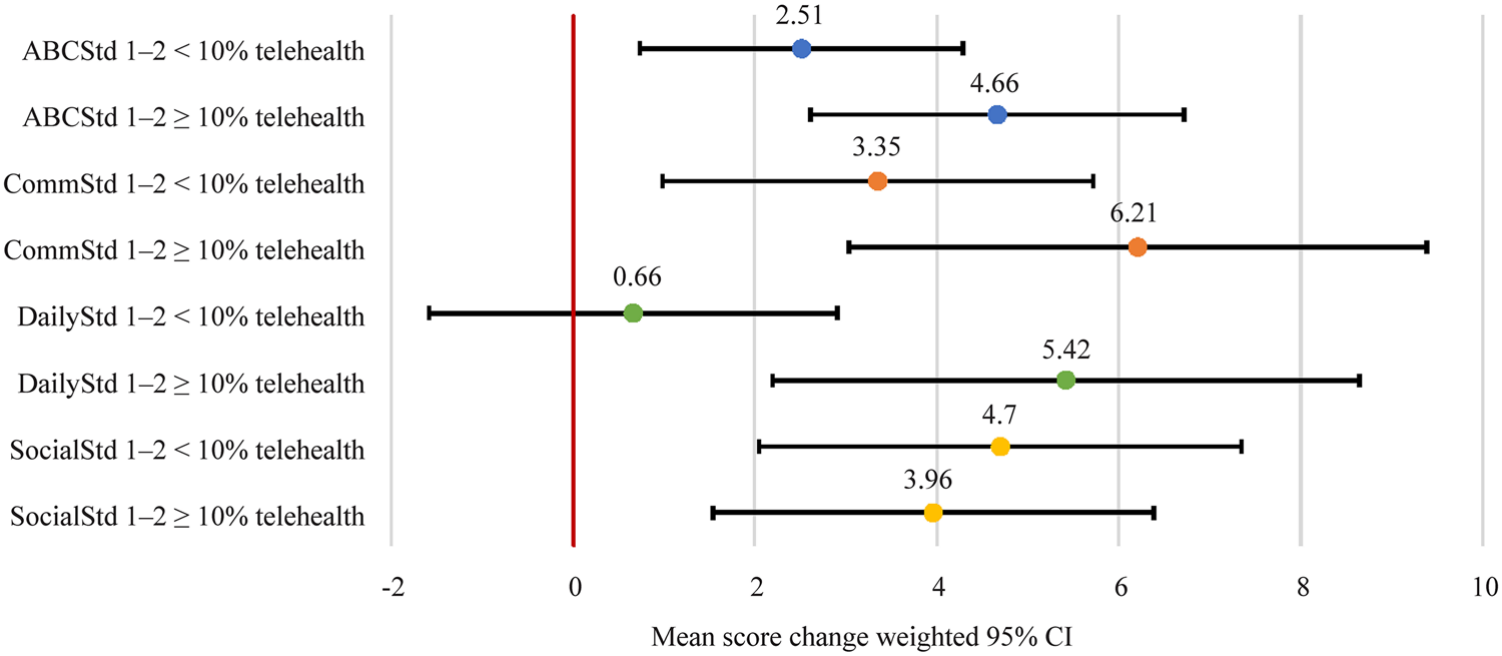
Differences in ABCStd score change relative to percent of BEH services delivered via telehealth. *ABCStd* standard Adaptive Behavior Composite score, *CommStd* communication domain score, *DailyStd* daily living domain score, *SocialStd* socialization domain score, *CI* confidence interval, *BEH* behaviorist

Children demonstrated statistically significant improvement (95% confidence intervals greater than zero) from time 1 to time 2 in ABCStd, CommStd, and SocialStd for both < 10% of BEH and ≥ 10% of BEH services delivered via telehealth (Fig. 3). In the case of DailyStd, those receiving < 10% of their BEH services via telehealth were not seen to have a statistically significant improvement in DailyStd scores, whereas those receiving ≥ 10% of their BEH services via telehealth did see a statistically significant improvement. Mean differences in the improvements of ABCStd, CommStd, DailyStd, and SocialStd scores with < 10% BEH vs. ≥ 10% BEH delivered via telehealth were insignificant at 2.148 (*P* = 0.123), 2.865 (*P* = 0.159), 4.759 (*P* = 0.019), and −0.743 (*P* = 0.686), respectively (Supplementary table 4).

Furthermore, there was no significant correlation between the average hours of BEH delivered via telehealth per month from Vineland 1 to Vineland 2 and change in ABCStd [Pearson correlation = 0.029 (*P* = 0.700)], CommStd [Pearson correlation = 0.078 (*P* = 0.304)], SocialStd [Pearson correlation =  −0.064 (*P* = 0.394)], and DailyStd [Pearson correlation = 0.060 (*P* = 0.429)] (Supplementary table 5). The absence of significant differences and correlations supports the notion that improvements in Vineland scores were independent of percent of BEH services delivered via telehealth, at least when the minority of BEH services were delivered virtually.

**Fig. 4:**
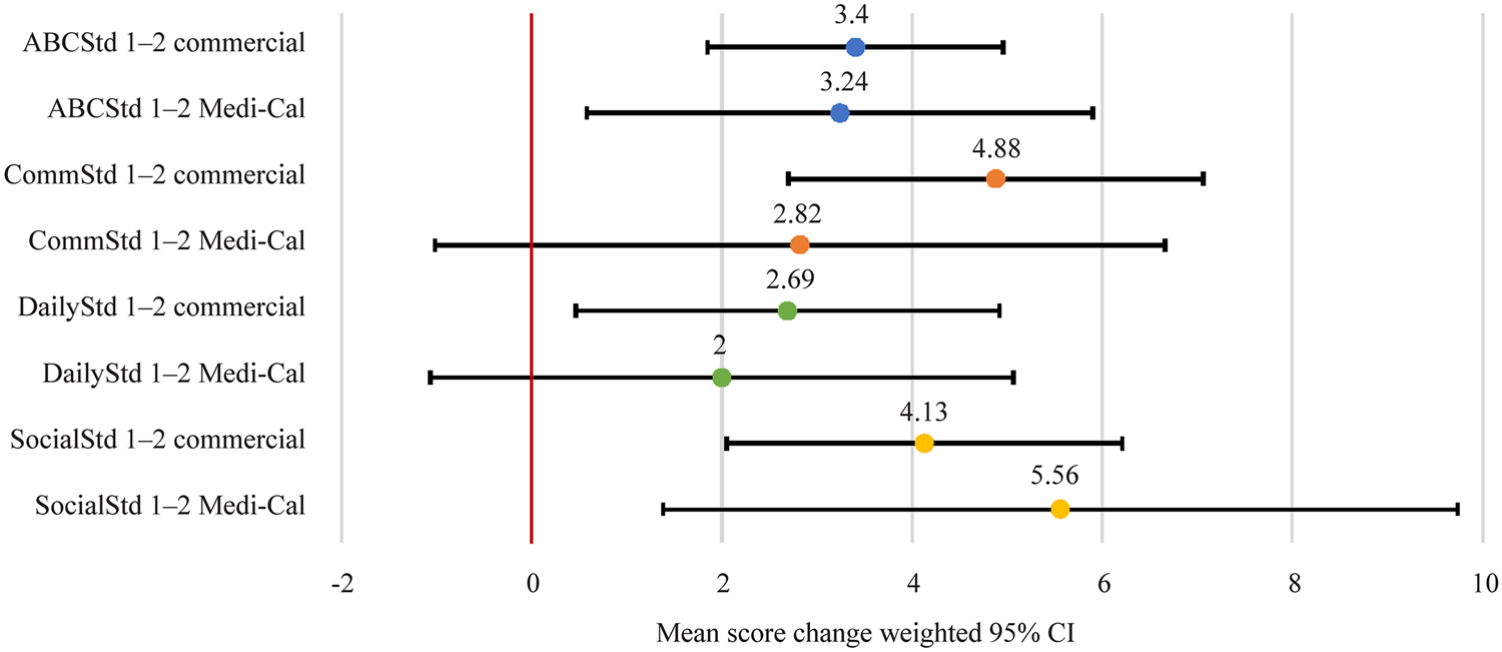
Differences in ABCStd score change relative to insurance type. *ABCStd* standard Adaptive Behavior Composite score, *CommStd* communication domain score, *DailyStd* daily living domain score, *SocialStd* socialization domain score, *CI* confidence interval, *Medi-cal* California’s Medicaid program

When segmenting based on payer type, children with commercial insurance demonstrated statistically significant improvement (95% confidence intervals greater than zero) from time 1 to time 2 in ABCStd as well as in all domain-specific Vineland scores (Fig. 4). Children with Medi-Cal had statistically significant improvements in ABCStd and SocialStd scores, whereas CommStd and DailyStd score improvements were not statistically significant. This is likely not a clinically significant distinction as the variation in score improvement amongst children with Medi-Cal was greater than that in children with commercial insurance owing to the small sample size. There were no statistically significant differences in the improvements of ABCStd, CommStd, DailyStd, and SocialStd scores with commercial insurance vs. Medi-Cal with mean differences of −0.167 (*P* = 0.916), −2.058 (*P* = 0.365), −0.694 (*P* = 0.720), and 1.427 (*P* = 0.552), respectively (Supplementary Table 6). The lack of significant differences supports the notion that improvements in Vineland scores were independent of insurance type.

## Discussion

The primary aim of this retrospective cohort study was to understand how a data-driven, client-centric ABA approach would impact the treatment-dose relationship for children with ASD. The results of the study support the hypothesis that children would have improved outcomes independent of the number of hours of service they received. This study suggests that the functional gains in the first year of care were clinically important. With well documented disparities in autism care [22], a particularly promising aspect of this study is that significant functional improvements were achieved independent of insurance type. Lower income Medi-Cal and higher income commercially-insured populations appear to have received equitable care.

While the data-driven, client-centric ABA intervention led to significantly improved outcomes, two different analyses suggest no association between these outcomes and the number of hours of service. Specifically, the Pearson correlations between service hours and Vineland standard scores were insignificant, and significant differences in improvement with respect to hours of service above or below 40 per month were absent. While these findings initially may seem to contradict prior research demonstrating a linear dose–response relationship, the interplay between these findings and prior research may be more nuanced.

Prior research has shown ambiguity in the dose–response relationship at higher doses, and at least 40% of the variation in treatment response has been shown to be attributable to factors unrelated to treatment dose [15]. The findings of this study may be explained by either a change in the dose–response curve and/or a change in the treatment response variance attributable to treatment intensity due to a fundamental change in care approach and most notably the process of ABA service hour allocation.

A key feature of the intervention being studied was rigorous, technology-enabled functional measurement that iteratively informed the care plan. The intervention had five components that may have contributed to a deviation from the traditional dose–response curve.

First, the intervention used a valid measure not just for reporting but also for clinical judgment. In the authors’ clinical experience, much of the ABA industry conducts Vineland assessments for insurance reporting and authorization purposes. Kyo made the Vineland a core aspect of the therapeutic approach and used it to inform care planning.

Second, the intervention relied on the use of HIT for real-time data insights to inform clinical decision making and population health management. Kyo made the decision to move to the online version of the Vineland and actively plugged that assessment into its data warehouse so that assessment data could be digitized and analyzed. Although the EMR and the analytics dashboard did not in and of themselves drive the improvements, the technology was instrumental in enabling the measurement processes to flourish. Given the historical dearth of HIT adoption among ABA providers, previously documented dose–response curves were likely constrained by ABA providers’ lack of technological tools that would allow them to titrate individualized treatment doses precisely.

Third, the intervention entailed rigorous adherence to processes that were outlined in a Clinical Policy Manual, which was routinely refined and was accompanied by learning and diffusion activities for staff.

The fourth attribute of the intervention that may have contributed to the improved outcomes independent of hours is the corporate culture that rewarded adherence to rigorous processes and client-centeredness. Kyo’s culture and processes dictate that care should be individualized for each child to account for individual differences, meaning that some children may not require intensive ABA care at all while other children may need lower doses of treatment and that treatment dose may change over time. In the authors’ experiences, some agencies only took clients that required at least 100 hours per month. Kyo did not exclude clients because of a minimum number of hours as that would not be client-centered. Additionally, if clients did not appear to require as many hours as previously needed, Kyo would reduce the requested hours for approval to the health plan.

Finally, a fifth variable that may have contributed to the functional improvements independent of hours is the nature of treatment provided. As previously stated above, the field of ABA stands with controversy given the variability in quality of ABA outcomes. ABA, when done poorly, has been described by some as leading to patients who are robotic in their interactions with others [23]. Others in the autistic community feel that the practice of reducing self-stimulatory behaviors is disrespectful of an individual’s right to self-determination [24]. These criticisms are generally specific towards ABA treatment that is adult-directed and that relies on Discrete Trial Training (DTT), a technique that stemmed from work in the 1970s and 1980s that focused on heavy repetition of skills and often relied on providing reinforcement unrelated to the task (e.g., delivering a preferred toy or an edible in response to answering questions). Some modern ABA providers, including Kyo, utilize more naturalistic, developmentally-mindful ABA treatment methods that are patient-centered, use natural reinforcers, and are implemented in a manner that is respectful of a patient’s autistic characteristics. Naturalistic developmental behavioral interventions (NDBIs), like the model espoused by Kyo clinicians, represent some of the most effective care currently available for patients with autism [25].

The secondary aim of this study was to evaluate the impact of the COVID-19-induced shift to telehealth clinical supervision on functional outcomes. The results of the study support the hypothesis that the modality of supervision, whether in-person or telehealth, would have no impact on outcomes, at least when the minority of supervisory services are delivered virtually.

When taken together with the treatment-dose relationship findings, the telehealth supervision findings may offer new opportunities for data-driven, client-centric ABA care to accelerate payment and delivery system reform in pediatric behavioral health. Almost all of pediatric behavioral health has been reimbursed on a fee-for-service (FFS) basis. While the broader healthcare industry has been shifting away from FFS and toward value-based payment (VBP), pediatric behavioral health has been constrained to FFS because of technology, operational competence, and financial incentive impediments [26]. The intervention studied here may promote ABA practice attributes that align clinical outcomes with financial incentives.

This study had several limitations. One limitation that is applicable to the broader ABA care space is that the Vineland assessment has limits in terms of evaluating the effectiveness of ABA care. First, the Vineland does not produce a direct measurement of behavior. Second, it is not considered to be a comprehensive measure of language or cognitive function. Third, while the Vineland is able to capture progress for clients who are closing the developmental gap, it does not adequately capture the socially significant progress that some children make with therapy, when the skills they learn do not translate into large domain gains on the Vineland. For example, a teen with ASD who engaged in self-injurious behavior of head banging who no longer engages in that behavior, who has learned to communicate “help”, “yes”, “no” and “stop”, and who has learned to dress themselves independently and in a year of care, might not show clinically significant progress on the Vineland. However, the skills learned during the course of treatment have a significant impact on the individual’s wellness and independence.

Other limitations of this study included that Kyo invested significant resources into developing its technology and data science capacity to enable a data-driven care model. Other ABA providers may not have the resources to make these types of investments in data infrastructure. Additionally, this study focused primarily on treatment intensity and did not explore total treatment dosage, which includes both intensity and duration. These data are limited by sample size and only show associations rather than causation, creating risk for confounding variables contributing to the observed improvements in outcomes. Future research is needed to verify these findings for reproducibility through prospective and randomized, controlled trials.

In conclusion, results of this study highlight the importance of treatment dosages that are customized to the individual receiving services. By iteratively tuning treatment dosage to the individual client’s needs, providers may be able to better maximize functional progress of the client, to preserve family time, and to utilize health plan dollars more efficiently. Given the proliferation of ABA providers in recent years and the variability in quality of ABA outcomes in the community setting, this study may reveal ways to avoid waste and abuse of care hours in the community. While many children were found to make progress with lower levels of care, some clients required higher levels. The results from this study should not discount the need for high treatment levels, known in the field as “comprehensive programs”, which entail 30 or more hours per week for some individuals with ASD. The diversity of needs among individuals with ASD calls for thoughtful customization of care plans including dosage and duration of care. Because the modality of supervision, whether in-person or telehealth, had no impact on outcomes, future care model design post-COVID-19 may consider more utilization of telehealth supervision to help improve access to care for clients that are difficult to reach in-person. The telehealth supervision findings taken together with the treatment-dose relationship findings could better align provider and payer objectives by moving pediatric behavioral health away from FFS and toward VBP through data-driven, client-centric ABA treatment.

## Supplementary Information

Below is the link to the electronic supplementary material.

Supplementary file1 (DOCX 13 KB)

Supplementary file2 (DOCX 16 KB)

Supplementary file3 (DOCX 17 KB)

Supplementary file4 (DOCX 16 KB)

Supplementary file5 (DOCX 16 KB)

Supplementary file6 (DOCX 16 KB)

## Data Availability

The data that support the findings of this study are available from Kyo but restrictions apply to the availability of these data, which were used under license for the current study, and so are not publicly available. Data are however available from the authors upon reasonable request and with permission of Kyo.
