# Supplementary material for: Data-driven, client-centric applied behavior analysis treatment-dose optimization improves functional outcomes
Source: World J Pediatr. 2022 Nov 17;19(8):753–60. doi: 10.1007/s12519-022-00643-0 (PMC9672611; doi:10.1007/s12519-022-00643-0)
Supplement: Supplementary file 1 — Supplementary file1 (DOCX 13 KB) [file 12519_2022_643_MOESM1_ESM.docx]

**Supplementary Table 1: Cohen’s D for Change in Standard Score Time 1 to 2**

|  | **Cohen's D** |
| --- | --- |
| **ABCStd1 vs ABCStd2** | 0.199 |
| **CommStd1 vs CommStd2** | 0.189 |
| **DailyStd1 vs DailyStd2** | 0.143 |
| **SocialStd1 vs SocialStd2** | 0.244 |
