# Supplementary material for: Data-driven, client-centric applied behavior analysis treatment-dose optimization improves functional outcomes
Source: World J Pediatr. 2022 Nov 17;19(8):753–60. doi: 10.1007/s12519-022-00643-0 (PMC9672611; doi:10.1007/s12519-022-00643-0)
Supplement: Supplementary file 2 — Supplementary file2 (DOCX 16 KB) [file 12519_2022_643_MOESM2_ESM.docx]

**Supplementary Table 2: Mean differences in change in Vineland scores from time 1 to 2 for < 40 vs ≥ 40 Hours of ABA services per month**

| **Independent Samples Test** | | | | | | | | | | | | |
| --- | --- | --- | --- | --- | --- | --- | --- | --- | --- | --- | --- | --- |
|  | | Levene's Test for Equality of Variances | | | t-test for Equality of Means | | | | | | | |
|  |  | F | Sig. | t | | df | Sig. (2-tailed) | Mean Difference | Std. Error Difference | 95% Confidence Interval of the Difference | |  |
|  |  |  |  |  |  |  |  |  |  | Lower | Upper |  |
| ABC1to2 | Equal variances assumed | 3.005 | .085 | -.785 | | 176 | .433 | -1.448 | 1.845 | -5.089 | 2.192 |  |
|  | Equal variances not assumed |  |  | -.664 | | 36.417 | .511 | -1.448 | 2.179 | -5.866 | 2.970 |  |
| Comm1to2 | Equal variances assumed | 4.828 | .029 | .159 | | 176 | .874 | .414 | 2.613 | -4.743 | 5.572 |  |
|  | Equal variances not assumed |  |  | .119 | | 34.010 | .906 | .414 | 3.475 | -6.647 | 7.476 |  |
| Daily1to2 | Equal variances assumed | .724 | .396 | -1.197 | | 176 | .233 | -3.081 | 2.574 | -8.160 | 1.998 |  |
|  | Equal variances not assumed |  |  | -1.169 | | 40.683 | .249 | -3.081 | 2.636 | -8.407 | 2.245 |  |
| Social1to2 | Equal variances assumed | .106 | .745 | -.538 | | 176 | .591 | -1.368 | 2.543 | -6.388 | 3.651 |  |
|  | Equal variances not assumed |  |  | -.543 | | 41.987 | .590 | -1.368 | 2.520 | -6.454 | 3.717 |  |
