# Supplementary material for: Data-driven, client-centric applied behavior analysis treatment-dose optimization improves functional outcomes
Source: World J Pediatr. 2022 Nov 17;19(8):753–60. doi: 10.1007/s12519-022-00643-0 (PMC9672611; doi:10.1007/s12519-022-00643-0)
Supplement: Supplementary file 3 — Supplementary file3 (DOCX 17 KB) [file 12519_2022_643_MOESM3_ESM.docx]

**Supplementary Table 3: Correlation between change in Vineland scores from time 1 to 2 and hours of BT, BEH, and PS**

| **Correlations** | | | | | | | | |
| --- | --- | --- | --- | --- | --- | --- | --- | --- |
|  | | ABC1to2 | Comm1to2 | Daily1to2 | Social1to2 | BTHrsV2 | BEHHrsV2 | PSHrsV2 |
| ABC1to2 | Pearson Correlation | 1 | .759^**^ | .768^**^ | .754^**^ | -.015 | -.005 | -.074 |
|  | Sig. (2-tailed) |  | .000 | .000 | .000 | .837 | .951 | .326 |
|  | N | 178 | 178 | 178 | 178 | 178 | 178 | 178 |
| Comm1to2 | Pearson Correlation | .759^**^ | 1 | .397^**^ | .368^**^ | .050 | .039 | -.117 |
|  | Sig. (2-tailed) | .000 |  | .000 | .000 | .510 | .607 | .119 |
|  | N | 178 | 178 | 178 | 178 | 178 | 178 | 178 |
| Daily1to2 | Pearson Correlation | .768^**^ | .397^**^ | 1 | .389^**^ | -.096 | -.084 | -.002 |
|  | Sig. (2-tailed) | .000 | .000 |  | .000 | .204 | .265 | .979 |
|  | N | 178 | 178 | 178 | 178 | 178 | 178 | 178 |
| Social1to2 | Pearson Correlation | .754^**^ | .368^**^ | .389^**^ | 1 | -.002 | .013 | -.067 |
|  | Sig. (2-tailed) | .000 | .000 | .000 |  | .980 | .862 | .375 |
|  | N | 178 | 178 | 178 | 178 | 178 | 178 | 178 |
| BTHrsV2 | Pearson Correlation | -.015 | .050 | -.096 | -.002 | 1 | .806^**^ | .172^*^ |
|  | Sig. (2-tailed) | .837 | .510 | .204 | .980 |  | .000 | .022 |
|  | N | 178 | 178 | 178 | 178 | 178 | 178 | 178 |
| BEHHrsV2 | Pearson Correlation | -.005 | .039 | -.084 | .013 | .806^**^ | 1 | .004 |
|  | Sig. (2-tailed) | .951 | .607 | .265 | .862 | .000 |  | .962 |
|  | N | 178 | 178 | 178 | 178 | 178 | 178 | 178 |
| PSHrsV2 | Pearson Correlation | -.074 | -.117 | -.002 | -.067 | .172^*^ | .004 | 1 |
|  | Sig. (2-tailed) | .326 | .119 | .979 | .375 | .022 | .962 |  |
|  | N | 178 | 178 | 178 | 178 | 178 | 178 | 178 |
| **. Correlation is significant at the 0.01 level (2-tailed). | | | | | | | | |
| *. Correlation is significant at the 0.05 level (2-tailed). | | | | | | | | |
