# Supplementary material for: Data-driven, client-centric applied behavior analysis treatment-dose optimization improves functional outcomes
Source: World J Pediatr. 2022 Nov 17;19(8):753–60. doi: 10.1007/s12519-022-00643-0 (PMC9672611; doi:10.1007/s12519-022-00643-0)
Supplement: Supplementary file 4 — Supplementary file4 (DOCX 16 KB) [file 12519_2022_643_MOESM4_ESM.docx]

**Supplementary Table 4: Mean differences in change in Vineland scores from time 1 to 2 for BEH delivered via telehealth < 10% vs ≥ 10%**

| **Independent Samples Test** | | | | | | | | | | |
| --- | --- | --- | --- | --- | --- | --- | --- | --- | --- | --- |
|  | | Levene's Test for Equality of Variances | | t-test for Equality of Means | | | | | | |
|  |  | F | Sig. | t | df | Sig. (2-tailed) | Mean Difference | Std. Error Difference | 95% Confidence Interval of the Difference | |
|  |  |  |  |  |  |  |  |  | Lower | Upper |
| ABC1to2 | Equal variances assumed | .314 | .576 | 1.531 | 176 | .128 | 2.148 | 1.403 | -.622 | 4.918 |
|  | Equal variances not assumed |  |  | 1.549 | 156.200 | .123 | 2.148 | 1.386 | -.591 | 4.886 |
| Comm1to2 | Equal variances assumed | .120 | .729 | 1.443 | 176 | .151 | 2.865 | 1.986 | -1.055 | 6.785 |
|  | Equal variances not assumed |  |  | 1.417 | 140.576 | .159 | 2.865 | 2.023 | -1.134 | 6.865 |
| Daily1to2 | Equal variances assumed | .014 | .907 | 2.450 | 176 | .015 | 4.759 | 1.943 | .925 | 8.593 |
|  | Equal variances not assumed |  |  | 2.372 | 133.419 | .019 | 4.759 | 2.006 | .791 | 8.727 |
| Social1to2 | Equal variances assumed | 3.941 | .049 | -.382 | 176 | .703 | -.743 | 1.945 | -4.582 | 3.096 |
|  | Equal variances not assumed |  |  | -.405 | 173.643 | .686 | -.743 | 1.835 | -4.364 | 2.878 |
