# Supplementary material for: Data-driven, client-centric applied behavior analysis treatment-dose optimization improves functional outcomes
Source: World J Pediatr. 2022 Nov 17;19(8):753–60. doi: 10.1007/s12519-022-00643-0 (PMC9672611; doi:10.1007/s12519-022-00643-0)
Supplement: Supplementary file 5 — Supplementary file5 (DOCX 16 KB) [file 12519_2022_643_MOESM5_ESM.docx]

**Supplementary Table 5: Correlation between change in Vineland scores from time 1 to 2 and share of BEH delivered via telehealth**

| **Correlations** | | | | | | |
| --- | --- | --- | --- | --- | --- | --- |
|  | | ABC1to2 | Comm1to2 | Daily1to2 | Social1to2 | BEHTeleShareV2 |
| ABC1to2 | Pearson Correlation | 1 | .759^**^ | .768^**^ | .754^**^ | .029 |
|  | Sig. (2-tailed) |  | .000 | .000 | .000 | .700 |
|  | N | 178 | 178 | 178 | 178 | 178 |
| Comm1to2 | Pearson Correlation | .759^**^ | 1 | .397^**^ | .368^**^ | .078 |
|  | Sig. (2-tailed) | .000 |  | .000 | .000 | .304 |
|  | N | 178 | 178 | 178 | 178 | 178 |
| Daily1to2 | Pearson Correlation | .768^**^ | .397^**^ | 1 | .389^**^ | .060 |
|  | Sig. (2-tailed) | .000 | .000 |  | .000 | .429 |
|  | N | 178 | 178 | 178 | 178 | 178 |
| Social1to2 | Pearson Correlation | .754^**^ | .368^**^ | .389^**^ | 1 | -.064 |
|  | Sig. (2-tailed) | .000 | .000 | .000 |  | .394 |
|  | N | 178 | 178 | 178 | 178 | 178 |
| BEHTeleShareV2 | Pearson Correlation | .029 | .078 | .060 | -.064 | 1 |
|  | Sig. (2-tailed) | .700 | .304 | .429 | .394 |  |
|  | N | 178 | 178 | 178 | 178 | 178 |
| **. Correlation is significant at the 0.01 level (2-tailed). | | | | | | |
